# Knock-out of vasotocin reduces reproductive success in female zebrafish, *Danio rerio*

**DOI:** 10.3389/fendo.2023.1151299

**Published:** 2023-08-21

**Authors:** Divya Ramachandran, Kusum Sharma, Vishal Saxena, Niepukolie Nipu, Dinusha C. Rajapaksha, Jan A. Mennigen

**Affiliations:** Department of Biology, University of Ottawa, Ottawa, ON, Canada

**Keywords:** nonapeptides, teleost, courtship behavior, ovary, CRISPR/Cas9

## Abstract

The vertebrate nonapeptide vasotocin/vasopressin is evolutionarily highly conserved and acts as neuromodulator and endocrine/paracrine signaling molecule. Circumstantial and mechanistic evidence from pharmacological manipulations of the vasotocin system in several teleost fishes suggest sex- and species-specific reproductive roles of vasotocin. While effects of vasotocin on teleost reproductive physiology involve both courtship behaviors and the regulation of the hypothalamic-pituitary-gonadal (HPG) axes, comprehensive studies investigating behavioral and physiological reproductive consequences of genetic ablation of vasotocin in a genetically tractable fish model, such as the zebrafish, are currently lacking. Here, we report the generation of homozygous CRISPR/Cas9-based vasotocin gene knock-out zebrafish. Breeding pairs of vasotocin knock-out fish produce significantly fewer fertilized eggs per clutch compared to wildtype fish, an effect coincident with reduced female quivering courtship behavior. Crossbreeding experiments reveal that this reproductive phenotype is entirely female-dependent, as vasotocin-deficient males reproduce normally when paired with female wild-type fish. Histological analyses of vasotocin knock-out ovaries revealed an overall reduction in oocytes and differential distribution of oocyte maturation stages, demonstrating that the reproductive phenotype is linked to oocyte maturation and release. Ovarian hormone quantification and gene expression analysis in mutant fish indicated reduced synthesis of Prostaglandin F_2α_, a hormone involved in ovarian maturation, egg release and regulation of female courtship behavior in some cyprinids. However, acute injection of vasotocin did not rescue the female mutant reproductive phenotype, suggesting a contribution of organizational effects of vasotocin. Together, this study provides further support for emerging roles of vasotocin in female teleost reproduction in an important teleost model species.

## 1 Introduction

The nonapeptide vasotocin is highly conserved in vertebrate evolution and is found in all non-mammalian vertebrates (1, 2). In its mature form, vasotocin forms a circular nonapeptide with a characteristic basic amino acid in position 8 (Cys-Tyr-Ile-Gln-Asn-Cys-Pro-Arg-Gly-NH_2_) which differentiates it from related oxytocin-lineage peptides considered to have evolved from the ancestral vasotocin gene at the base of vertebrate evolution (1, 2). The vasotocin gene encodes a precursor protein whose cleavage generates the mature nonapeptide, a carrier protein termed neurophysin 2, as well as a peptide fragment termed copeptin (1, 2). In zebrafish and other teleost fishes studied to date, vasotocin is produced in giganto-, magno-, and parvocellular neurons of the preoptic area (POA), from where it innervates diverse hypothalamic regions and extrahypothalamic brain regions (3–8). Innervation of extrahypothalamic regions includes sensory regions (9, 10) and hindbrain motorneurons (8, 11). These regions are directly involved in the perception of environmental stimuli relevant to reproduction on the one hand, and in the control of courtship behavior on the other. From the POA, vasotocinergic innervation also robustly extends to the pituitary gland, especially in the *pars nervosa*, where the nonapeptide is released into circulation via terminal buttons (12, 13). Albeit to a lesser extent, vasotocinergic innervation has also been described to contact gonadotrophs at least in some teleost species (12, 14). In the few teleost fishes investigated, peripheral vasotocin expression has been reported in the testes (15) and the ovary (16, 17). Altogether, the (neuro)anatomy of the vasotocin system suggests possible roles in regulating behavioral and endocrine components of reproduction in teleost fishes by acting as central neuromodulator and/or endocrine or paracrine factor along the hypothalamus-pituitary-gonadal (HPG) axis.

Indeed, circumstantial as well as functional evidence from pharmacological, and more recently, genetic ablation studies in different teleost fishes support this notion. For example, reproductive stage-dependent vasotocin expression and/or translated vasotocin protein levels in the whole brain, circulation and the ovary have been reported in several fish species, including the round goby *N. melanostomus* (18, 19), the three-spined stickleback *G. aculeatus* (20, 21), and the Asian stinging catfish, *H. fossilis* (16). Functionally, pharmacological studies (22–25) have implicated vasotocin in stimulating male courtship behavior in several teleost fish species, including the zebrafish, *D. rerio* (26). Genetic ablation of the vasotocin gene in male Japanese medaka, *O. latipes* resulted in decreased sexual motivation and disrupted mate-guarding behavior (27). Evidence from investigations of vasotocin peptide effects on courtship behavior in the peacock blenny, *S. pavo* demonstrate stimulatory effects in females and sneaker males but not dominant males (28), underlining the importance of considering possible sex- and species-specific effects of this nonapeptide between teleost fishes. Regarding the HPG axis, studies in teleost species such as the sailfin molly *P. latipinna* (13), the stinging catfish (29), and the cichlid *C. dimerus* (15) unequivocally point to hypophysiotrophic roles of vasotocin in the stimulation of LH and FSH subunit expression and/or LH release. Finally, endocrine and/or paracrine roles for vasotocin have been reported to regulate gametogenesis, steroidogenesis and gamete release in male and female teleosts. Regarding effects of vasotocin on male gonads, testes incubated with vasotocin *in vitro* stimulated testosterone (T) release in both rainbow trout, *O. mykiss* (30) and the cichlid chanchita, *C. dimerus* (15). Administration of vasotocin in the catfish, *C. magur*, increased spermatozoa concentration in strippable milt (31). Insights into the role of vasotocin in the regulation of female gonadal function stem largely from a suite of studies conducted in the Asian stinging catfish (32–37). In this species, vasotocin has been shown to promote estradiol (E_2_) production in previtellogenic follicles and to promote final oocyte maturation via steroidogenic shift and characterized by an inhibition in E_2_ but induction of maturation-inducing steroid and progestin (P_4_) in post-vitellogenic follicles to promote germinal vesicle breakdown (38). Finally, vasotocin has also been shown to contribute to oocyte hydration via aquaporin 1ab (*aqp1ab*) transcript abundance (38).

While these studies reveal that vastocin generally plays important stimulatory roles in reproduction of teleost fishes, its role in zebrafish, a genetically and increasingly physiologically tractable model species to investigate reproductive function (39) is not well understood (26). Importantly, in the reproductively diverse infraclass of teleost fishes, it has been postulated that sociosexual nonapeptide function is likely species-specific, creating a clear need for comparative studies (38, 40). Finally, few, if any, previous studies, including recent genetic ablation studies in Japanese medaka (27), have probed vasotocin function in teleost reproduction comprehensively *in vivo* by investigating both behavioral and HPG axis consequences in a single species. This is, however, important, as a clear link between gamete production capacity and reproductive behavior has been demonstrated in medaka knock-out fish (41), and functional organismal consequences of vasotocin-dependent regulation of gametogenesis and steroidogenesis in tissues such as in the ovary (38), remain to be evaluated *in vivo*.

To address these gaps and to test the hypothesis that vasotocin contributes to reproductive success in zebrafish in either or both sexes through regulation of courtship behavior and/or regulation of the HPG axis, we here report the creation of a homozygous zebrafish vasotocin knock-out model (*avp* ^-/-^). Following the identification of a female-specific impairment of reproductive function, we subsequently assessed the potential mechanistic basis of this phenotype by assessing ovarian function using histological, biochemical, and molecular experimental approaches.

## 2 Materials and methods

### 2.1 Animals and experimental design

All experiments were carried out in accordance with animal care guidelines provided by the Canadian Council on Animal Care and with prior approval from the University of Ottawa Animal Care Committee (Protocol #BL-3561). Wild-type (WT) zebrafish were sourced from the in-house AB strain stock at the University of Ottawa and maintained in 12 l tanks at a density of 3 fish/l in a recirculating system (Techniplast, Montréal, QC, Canada). The system was supplied with salt-dosed (Instant Ocean, St. Blacksburg, VA, USA) RO water (hereafter “system water”) maintained at a pH of 7.3, a conductivity of 400 μS, and a temperature of 28°C under a 14:10 h light–dark cycle. Following transition to exogenous feeding, larval fish were fed twice daily with different-sized GEMMA zebrafish diets (Skretting, Vancouver, BC, Canada) according to life-stage. Adult fish were fed a mixed diet (Adult Zebrafish diet, Zeigler Bros Inc, Gardners, PA, USA; Larval AP-100, Zeigler Bros Inc, Gardners, PA, USA; Golden Pearls, Artemia International, Fairview, TX, USA) twice daily. Embryos (first for the development of the *avp* ^-/-^ knock-out line and later for maintenance of WT and *avp* ^-/-^ lines to supply animals for experiments) were attained by pairing individual male and female fish (4-8 months post-fertilization; mpf) previously separated by visual inspection of pectoral fin breeding tubercles, body shape and coloring (42, 43), and maintained in single sex groups. Individual males and females were then moved to 1 l static breeding tanks with a perforated base insert and separated with a removable divider overnight. The following morning at the beginning of the light cycle, the barrier was removed, fish allowed to interact for 2 h, and eggs collected after spawning.

### 2.2 Generation of avp ^-/-^ knock-out zebrafish

**Figure 1:**
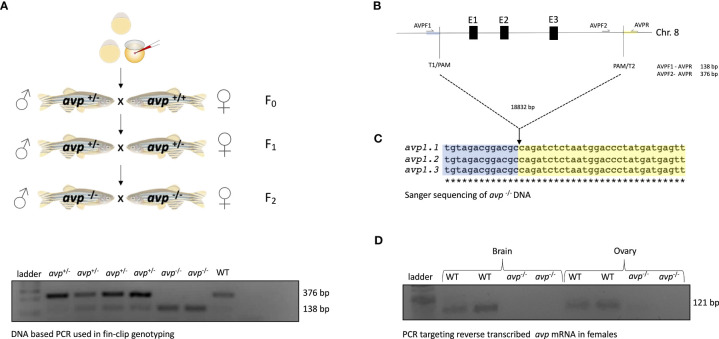
Generation and validation of *avp ^-/-^* zebrafish: **(A)** Microinjection and breeding scheme. **(B)** CRISPR/Cas9 construct and targeted locus. **(C)** DNA sequencing of *avp* locus in *avp* ^-/-^ fish **(D)** Agarose gel electrophoresis analyzing *avp ^-/-^* transcript specific amplicons in brain and ovaries obtained by RT-PCR. Please refer to the text for detailed explanations.

A current zebrafish genome annotation (GRCZ11) in Ensembl was used as reference for zebrafish gene coding and transcript information. For sgRNA design, CHOPCHOP software was employed (44). A protocol published by Gagnon et al. (45) was followed to generate templates for sgRNA transcription by annealing gene specific oligonucleotides containing the T7 promoter sequence, the 20 base pairs (bp) target site (**Table 1**) followed by the PAM sequence, and a complementary region of 80 bp constant oligonucleotide. We synthesized two sgRNAs to be injected simultaneously to achieve the deletion. All sgRNAs were transcribed using the HighScribe™ T7 Quick High Yield RNA synthesis kit (New England BioLabs, Ipswich, MA, USA), and the resulting RNA was purified using 5 M ammonium acetate and ethanol precipitation. RNA bands were detected by electrophoresis on a 1% agarose gel and RNA purity and concentration were measured using NanoDrop 1000 spectrophotometer (Thermo Fisher Scientific, Waltham, MA, USA). The CRISPR/Cas9 based approach (**Figure 1A**) was designed to completely delete the *avp* ^-/-^ gene locus (NCBI Gene ID: 352922, genomic region NC_007119.7, Chr. 8), excising a region of 18832 nt including all three *avp* exons (**Figure 1B**).

**Table 1:** CRISPR/Cas9 construct genome target sequences, DNA genotyping and transcript primer sequences.

Sequence ID	Target and location according to NCBI zebrafish genome GRCz11 Primary Assembly	Sequence
T1/PAM	Genomic downstream of *avt* locus NC_007119.7:1144047	GACATGTAGACGGACGC*CAG/GGG
PAM/T2	Genomic upstream of *avt* locus NC_007119.7:116359	CCA/GGA*CAGATCTCTAATGGACC
AVTF1	Genomic upstream of *avt* locus NC_007119.7:1143996	AGGATCTGTGACTACGCAATC
AVTF2	Genomic upstream of *avt* locus NC_007119.7:116250	AGTGACGTGTCAACACTAGT
AVTR1	Genomic upstream of *avt* locus NC_007119.7:116405	GTAATGCGGATAACTCTCAGG
AVTFW	*avp* mRNA NM_178293.2 nucleotides 108-128	CCCAGCCGGAGCCCATCAGA
AVTRV	*avt* mRNA NM_178293.2 nucleotides 219-239	CCATGCAGACCTGCGCCTCC

Briefly, microinjections were performed in 1-cell stage embryos (F0). The injection solution comprised of 40 ng/μl for each sgRNA, 20 ng/μl Cas9 protein (New England BioLabs, Ipswich, MA, USA), and 0.1% Phenol Red (Sigma, Burlington, MA, USA) suspended Danieau buffer (in mmol/L: 58 NaCl, 0.7 KCl, 0.4 MgSO_4_, 0.6 Ca(NO_3_)_2_, and 5.0 Hepes; pH 7.6). Potential founders were identified by consistent appearance of *avp* ^+/-^ genotypes in fin clips of a fraction of F1 offspring obtained from crosses of adult (90 dpf) F0 and WT fish. A mutant line was then established by incrossing heterozygous F1 *avt* ^+/-^ carrying the same deletion to generate homozygous F2 *avp* ^-/-^, again assessed by fin clip genotyping (**Figure 1A**). In all cases, fin clips were collected following brief anesthesia in buffered tricaine mesylate (MS-222; Syndel Laboratories, Nanaimo, BC, Canada; 100 mg/l in 20 μl of 50 mmol/l NaOH at 95°C for 10 min followed by neutralization with 2 μl of 1 mol/l l Tris–HCl (pH 8). The PCR amplification reaction mix consisted of 1 μl of fin clip DNA digest template in 24 μl PCR reaction master mix, which contained 12.5 μl 2X GoTaq Green Master Mix (Promega, Madison, WI, USA), 1 μl forward primer 1, 1 μl forward primer 2, 1 μl reverse primer (all IDT, Coralville, IA, USA, **Table 1**) and 8.5 μl ddH_2_O. The PCR reactions were run with a temperature cycle of 94°C for 3 min followed by 35 cycles of 95°C for 30 s (denaturation), 60°C for 30 s (annealing) and 72°C (extension) for 1 min. Following the last cycle, a final 72°C step was run for 10 min and samples maintained at 4°C until analysis by gel electrophoresis. Gel electrophoresis using a 1% Agarose gel containing Red Safe dye (Froggabio, North York, ON, Canada) was run for 30 min at 100 V to visualize amplicons. Using the three primers listed in **Table 1**, this reaction produced a single 376 bp band for WT, two bands (376 bp and 138 bp) for heterozygous *avp* ^+/-^ mutants, and single band (138 bp) for homozygous *avp* ^-/-^ mutants (F2). The PCR products containing amplicons from fin clips of fish carrying the homozygous deletion of all three *avp* exons were sent for Sanger sequencing (Genome Quebec, McGill University, Montreal, Canada), and alignment with the wild-type genomic sequence confirmed an 18832 bp deletion encompassing all three *avp* exons (**Figure 1C**) Finally, absence of the *avp* mRNA transcript was confirmed in adult F2 *avp* ^-/-^ using total RNA extraction from brain and ovary using the Trizol Reagent (Invitrogen, Burlington, ON, Canada), followed by cDNA synthesis using the Quantitect reverse transcriptase kit (Qiagen, Hilden, Germany) according to the manufacturers’ instructions. cDNA synthesized from total RNA extracted from brain and ovaries of WT and *avp* ^-/-^ was then used as template in PCR reactions prepared and run as described for fin clip DNA analysis with the exception that 1 μl cDNA template from brain or ovary was used in a master mix consisting of 1 μl *avp* FW, 1 μl *avp* RV primer (IDT, **Table 1**), 12.5 μl 2X GoTaq Green Master Mix (Promega) and 9.5μl ddH_2_O. Gel electrophoresis using a 1% Agarose gel containing Red Safe dye (Froggabio, North York, ON, Canada) was run for 30 min at 100 V to visualize 121 bp *avp* transcript amplicons (**Figure 1D**).

#### 2.2.1 Experimental design

**Figure 2:**
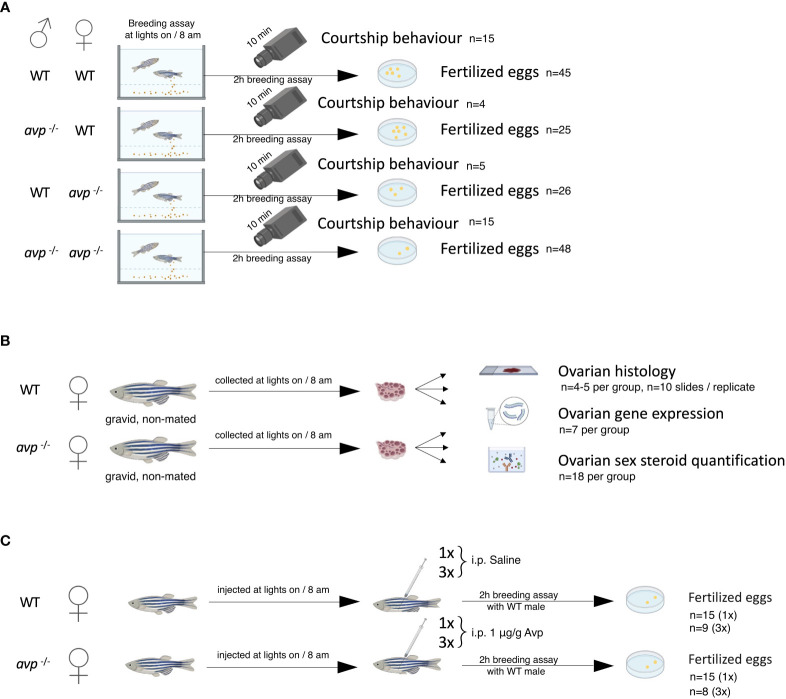
Schematic representation of experimental design: **(A)** Breeding assays. **(B)** Investigation of mechanistic basis of the female reproductive phenotype using ovarian histology, quantification of ovarian hormone concentrations, and ovarian gene expression assays. **(C)** Single (1x) and repeated (3x over a period of 9d prior to breeding assay) vasotocin injection in *avp* ^-/-^ females prior to breeding to assess possible rescue of the reproductive phenotype. Please refer to the text for detailed explanations.

The reproductive phenotype and potential underlying mechanisms were assessed using a three-tiered approach (**Figures 2A–C**). First, reproductive success was assessed in breeding assays using all four genotype combinations of male and female WT and *avp* ^-/-^ zebrafish (**Figure 2A**). Reproductive success in breeding assays was assessed as percentage of pairs successfully producing fertilized eggs and quantified by counting the number of fertilized eggs per breeding pair. Because very few non-fertilized eggs occurred in very few breeding pairs of different genotype combinations, fertilized eggs numbers were not significantly different from total egg numbers (data not shown). Following the identification of a female-specific phenotype, underlying mechanisms were assessed at the level of the ovary using histology, hormone quantification, and gene expression assays (**Figure 2B**). Finally, rescue experiments were conducted to assess whether activational and/or organizational effects of vasotocin contribute to the observed phenotype (**Figure 2C**). In all cases adult WT and mutant zebrafish were 6-9 months post fertilization (mpf).

#### 2.2.2 Zebrafish breeding assays, quantification of reproductive success, and courtship behavior

Single adult male and female WT and male and female *avp* ^-/-^ zebrafish (6-9 mpf) were combined as breeding pairs in new 2 l breeding tanks to assess reproductive success. Following the observation of decreased reproductive success in *avp ^-/-^* breeding pairs compared to wildtype breeding pairs, additional breeding pairs in which WT of one sex was paired with the *avp* ^-/-^ fish of the other sex were investigated to assess sex-specific contributions of vasotocin to reproductive success in zebrafish. Thus, four groups were investigated (**Figure 2A**): male WT x female WT (n=45), male *avt* ^-/-^ x female WT (n=25), male WT x female *avt* ^-/-^ (n=26) and male *avt* ^-/-^ x female *avt* ^-/-^ (n=48). While all WT and *avp* ^-/-^ animals used in breeding assays were derived from same-sex housed stock, each individual fish was used in a single breeding assay and thus genotype combination group. Pairs received additional feeding in the afternoon the day before the mating assay and were separated by divider overnight. The following day, the divider was removed at the onset of the light cycle (8:00 h). Breeding pairs were allowed to interact for 2 h in the Zebracube and Zebrafish Behavior platform (Viewpoint, Montréal, QC, Canada), allowing for a controlled environment. For a subset of breeding pairs, videos of courtship behaviors were recorded using a Dragonfly2 DR2-HIBW camera with 30 frames/s (Point Grey Research, Richmond, BC, Canada). Following the 2 h breeding period, eggs, if present, were collected using a strainer and placed in a petri dish filled with methylene blue system water. The total number of fertilized eggs (translucent, symmetrical with increased perivitelline space) were then quantified as measure of reproductive success. If present, unfertilized eggs (slightly yellow in color and with granular appearance) and/or dead eggs (white in color and often broken down) were counted separately and removed from the petri dish. Following consistent demonstration that irrespective of genotype, all analyzed courtship behaviors declined after initial interaction, the first 10 min of interaction were analyzed for each video in a subset of animals for which the interaction was recorded. Specifically, and as previously described (26, 46, 47), the number of individual male-initiated chasing events, the total duration of these chasing events, the number of nudging (targeted physical contacts of the male’s head with the female flank), encircling (male swimming a full close circle around the female), and quivering events (close flank by flank contact and parallel swimming of male and female in a jagged line, followed by female flexing away from the male) were analyzed by an experimenter blind to the crossed genotypes.

#### 2.2.3 Investigation of the mechanistic basis of the reproductive phenotype

##### 2.2.3.1 Ovarian histology

The gonadosomatic index (GSI) was assessed from n=25 WT and n=26 *avp* ^-/-^ fish (6-9 mpf) sampled from female holding tanks not used for courtship assays. Following terminal anesthesia of WT and *avp ^-/-^* females at the onset of lights in the morning, the fish were gently dried on paper towel, and weighed. Abdominal sections containing the ovary were then collected from WT and *avp* ^-/-^, and ovaries carefully dissected and weighed. The GSI was calculated by dividing the ovarian mass by total body mass. Each section was placed in a 1.5 ml pre-weighed Eppendorf tube and the weight recorded. For n=4 WT and n=5 *avp* ^-/-^ ovaries, whole fish abdominal samples were placed in 4% PFA PBS solution and incubated at 4°C overnight and washed for 3 x 15 min in PBS the following day. The samples were subsequently transferred to a 0.5 M EDTA (pH= 8.0) solution to decalcify tissue for one week at 4°C. The following week, the samples were washed for 3 x 15 min in PBS, before successive dehydration steps in 10%, 20% and 30% sucrose in PBS solutions. Samples were placed in each sucrose solution until samples sunk. Finally, samples were embedded in molds with OCT (Thermo Fisher Scientific) and stored at -80°C until sectioning. Each sample was sectioned across the sagittal plane using a Cryostat (LEICA CM 3050 S, Leica, Concord, ON, Canada) and serially cut into thirty (10 slides with 3 sections each per individual ovary) 12 μm sections (OT = -20°C, CT = -26°C), which were then collected on Fisherbrand Superfrost Plus microscope slides (Thermo Fisher Scientific). All slides were subsequently rinsed in 1 x PBS for 3 min, then placed under a gentle stream of tap water for 3 min to wash off the OCT. For H&E staining, the slides were incubated for 30 s in Mayer’s Hematoxylin, then placed in 1 x PBS for 20 seconds and washed under a gentle stream of tap water for 1 min. The slides were then dipped in 70% and 95% EtOH for 30 s consecutively and placed in the counterstain Alcoholic Eosin for 30 s. Once removed, the slides were dehydrated through 2 x 15 seconds in 95% EtOH and 3 x 15 seconds in 99% EtOH. To ensure the tissue was cleared of any other residue, slides were immersed in xylene. 3 x for 1 min. Ovary sections were finally imaged using a dissection microscope (Olympus SZ2-ILST, Olympus, Richmond Hill, ON, Canada) using a 6.7 x magnification to determine the number of eggs and largest diameter of each oocyte. Additional images of representative slides were taken using a (CX41 with U-CAMD3, Olympus), at 40x magnification. All images were acquired using the Infinity II microscopy camera (Teledyne Lumenera, Ottawa, ON, Canada) and images were analyzed using ImageJ (www.https://imagej.nih.gov/ij/). Briefly, the total number of oocytes per section and size-based oocyte stages (I-V) were determined for each of the eggs measured according to Li and Ge (39). For all non-circular oocytes, the largest diameter was measured. All oocytes with visible histological artifacts were eliminated from analyses, and redundant measurements of oocytes avoided by labelling counted oocytes in ImageJ using the numbering tool. Oocyte stages were expressed as percentage of total oocytes within WT or *avp* ^-/-^ ovaries respectively.

##### 2.2.3.2 Quantification of ovarian reproductive hormone concentrations

Whole ovaries from adult WT (n=18) and *avp* ^-/-^ (n=18) female zebrafish (6-9 mpf) held in all female tanks not used for breeding assays were carefully extracted following terminal anesthesia and weighing of the whole animal as previously described. All animals were collected upon onset of lights in the morning. Tissues were then weighed, placed in 1.5ml microcentrifuge tubes, and stored -80°C until processing. To extract ovarian hormones, ovarian tissue samples were thawed on ice and 200 μl ELISA buffer (Cayman Chemicals, Ann Arbor, MI, USA) was added to each tube. The samples were then sonicated, ensuring the probe was cleaned with 75% EtOH and RNAse free water in between each sample. Following homogenization, 1 ml of diethyl ether was placed into each sample, vortexed and allowed to sit for 30 min. The samples were then centrifuged at 3000 g for 5 min and flash frozen at -80°C for 30 min. The liquid phase was removed and placed into new microcentrifuge tubes. The ether was evaporated under a gentle stream of nitrogen under the fume hood, and the extraction steps were then repeated two more times. After the final extraction step, 250 μl extraction buffer was added to the tubes, vortexed and placed in a heat block for 5 min at 65°C. The samples were vortexed, placed on the heat block for additional 5 min and vortexed for a final time. The extracts were then placed at -80°C until use. Extracts were used for the quantification of 17-β estradiol (E_2_), Progesterone (P_4_) and Prostaglandin F_2α_ (PGF_2α_) using ELISA kits #501890, #582601 and #516011 (all Cayman Chemicals) according to manufacturer’s instructions. Assay sensitivities (defined as 80% B/B_0_) were 9.43 pg/ml for E_2_, pg/ml 7.65 pg/ml for P_4_ and 19.20 pg/ml PGF_2α_, respectively. All samples were run in duplicate and a cut-off of <20% intra-assay CV was applied for replicates. Average intra-assay CVs were 13.96% for the E_2_ assay, 15.75% for the P_4_ assay, and 13.4% for the PGF_2α_ assay. All analyte concentrations were corrected for sample dilution factors and normalized by ovarian weight prior to analysis.

##### 2.2.3.3 Ovarian gene expression assays

Ovaries from female WT (n=7) and *avp ^-/-^* mutants (n=7) were collected from all female housing tanks when lights were turned on in the morning and immediately stored at -80°C until processing as previously described. To extract total ovarian RNA, frozen tissues were homogenized in 250 μl of TRIzol reagent (Invitrogen) using a sonicator. The probe was cleaned with 75% EtOH and RNAse-free water in between each sample to avoid cross-contamination. Total RNA was extracted according to the manufacturer’s instruction and the total RNA pellet resuspended in 30 μl of DEPC water. The purity and concentration of total RNA was then assessed using a NanoDrop and was used to generate cDNA. cDNA was synthesized using the QuantiTech Reverse Transcription Kit (Qiagen) according to the manufacturer’s instructions, using 1 μg of total RNA per sample. Controls devoid of reverse transcriptase enzyme were used to monitor potential contamination during subsequent gene expression assays. All samples were stored at -20°C until used as templates in gene expression assays.

Two-step SYBR green-based, semi-quantitative real-time RT polymerase chain reaction (RT-qPCR) was used to quantify relative fold-changes of 11 ovarian gene transcripts with known function in different stages of zebrafish oocyte maturation, as reviewed by Li and Ge, 2020 (39). Briefly, the assay profiled the expression of two genes enriched in germ cells (*vasa*, *nanos2*), the gonadotropin receptors (*lhcgr*, *fshr)*, the lipoprotein *vtg1*, nuclear and membrane receptors for steroids (*ar*, *pgr*, *pgrmc1*, *pgrmc2*) and genes involved in PGF_2α_ synthesis pathway (*pla2g4ab*, *ptgs2*). A complete list of gene IDs, full gene names, primer sequences used in gene expression assays and annealing temperatures can be found in **Table 2**. All assays were run on a BioRad CFX96 machine (Bio-Rad, Mississauga, ON, Canada) and consisted of a serially diluted standard curve consisting of pooled cDNA, a negative no-RT control, and individual samples run in duplicates. For each sample, the total reaction volume was 20 μl, which consisted of 1 μl of diluted cDNA template, 1 μl of 10 nM specific forward and 1 μl of 10 nM specific reverse primer (**Table 2**), 10 μl of SsoAdvanced Universal Inhibitor-Tolerant SYBR Green Supermix (Bio-Rad) and 7 μl of DEPC-water. For each assay, parameters were an initial two min activation step at 95 °C, followed by 40 cycles consisting of a 20 s denaturation step at 95 °C combined with a 30 s annealing and extension step at a primer specific temperature between 58-63 °C. After each run, melting curves were produced by gradually increasing the temperature and final curves were assessed for single peaks to confirm specificity of the reactions. The acceptable range for amplification efficiency calculated from serially diluted curved was 90-110%, with an R^2^ > 0.95. Relative gene expression was subsequently normalized using the NORMA-gene method (48) and relative transcript changes normalized to WT control zebrafish to determine relative fold-change for a given transcript in the *avp* ^-/-^ compared to WT.

**Table 2:** Profiled transcripts, primer sequences, and real-time RT-PCR reaction conditions used to quantify gene expression in WT and *avp* ^-/-^ ovarian tissue.

Gene symbol	NCBI Gene ID	Gene name	Forward Primer (5’3’)	Reverse Primer (3’5’)	Annealing temperature (°C)	Efficiency (%)	R^2^
*ddx4*	30263	DEAD (Asp-Glu-Ala-Asp) box polypeptide 4/Vasa	GCTGCAATGTTCTGTGTGCT	CATGAGGGTTTGTCGTTCCT	60	111.4	0.98
*nanos2*	101885179	nanos homolog 2	ACGGCTGTTTCCTGATGTG	CCCTTGCCTTTAGTCTGTGG	58	104.9	0.96
*lhcgr*	402920	luteinizing hormone/choriogonadotropin receptor	AAAAGGACGAGTCGCTGAAA	AAAACAAGAAGCAGGGCAGA	60	92.7	0.96
*fshr*	195820	follicle stimulating hormone receptor	TACCCCATCAATTCCTGCTC	CATCCAGATTCCACGCTTTT	60	99.3	0.92
*vtg1*	559475	vitellogenin 1	TTCAGACCCCCATTCAACTC	TTTCTCCAAGGAGGCAACAC	60	107.4	0.99
*ar*	100005148	androgen receptor	AGGGAGTTTTCCGACGAGTT	TTGGCAGGGTAAAAGTGAGG	60	111.08	0.97
*pgr*	569575	progesterone receptor	GAGTCCTTCGCTGATGTTCC	CTCTGGCTGTGTGTTGTCGT	58	95.2	0.99
*pgrmc1*	10857	progesterone receptor membrane component 1	CCTGGCTACGTTCTGTTTGG	GGGTCCGCTCTAATCCTTCT	58	112.7	0.99
*pgrmc2*	406378	progesterone receptor membrane component 2	TTCACGTCTGTGAGCGAAAC	AGAGGGAAACGGATGGAAAC	58	108.2	0.99
*pla2g4ab*	559087	phospholipase A2, group IV Ab (cytosolic, calcium-dependent)	ACAGGTGAACAAGGGCAGAG	ACAGGTGAACAAGGGCAGAG	60	96.5	0.98
*ptgs2a*	246227	prostaglandin-endoperoxide synthase 2a	GAGCTTCTCACACGCATCAA	ATGGGACCTTGACAACAGGA	63	86.4	0.98

#### 2.2.4 Vasotocin injection rescue experiments

Acute single and repeated intraperitoneal (i.p.) vasotocin (Cayman Chemicals, purity > 95%) experiments were conducted in female mutant fish not previously used in breeding assays to assess whether the observed female reproductive phenotype in *avp* ^-/-^ zebrafish could be rescued through activational effects of vasotocin (**Figure 2**). For the single injection experiment, female *avp ^-/-^* zebrafish were separated and held in single-sex tanks for two weeks prior to the start of the experiment. At the onset of the light the next morning, female *avp* ^-/-^ fish were anesthetized using 0.24 mg/ml of tricaine (Syndel Laborotories) and once deep anesthesia was confirmed, i.p. injected with either physiological saline (n=15) or 1 μg/g bw vasotocin (n=15) in physiological saline. Following ~10 min recovery from anesthesia, female fish were set up for breeding with WT male zebrafish and mating success quantified as previously described. Using the same experimental parameters, a second repeated injection rescue experiment was conducted using *avp* ^-/-^ zebrafish (n=9 saline injected; n=8 vasotocin injected) spawned at the beginning of the experiment to release of residual eggs and subsequently injected on day 3, 6 and 9 after before quantification of reproductive success on the morning of day 10. To assess the possibility that i.p. injection itself may represent a physiological stressor sufficient to inhibit female reproductive success as measured by egg fertilization following breeding, a group of WT females was i.p. injected with physiological saline but did not exhibit different breeding success compared to un-injected WT females assessed in breeding assays as described previously (data not shown).

### 2.3 Determination of pleiotropic effects in avp knock-outs

Given the reported role of Avp in several aspects of teleost physiology known to affect reproduction, we sought to assess the potential for altered somatic growth and basal endocrine stress axis activity in *avp* ^-/-^ knock-out to affect reproductive physiology.

#### 2.3.1 Quantification of somatic growth in juveniles

To assess indices of somatic growth up to adulthood (3 mpf), body mass and body length (measured as fork length) in WT (n=20) and *avp ^-/-^* mutants (n=20) were analyzed at 30, 60 and 90 dpf. Each fish was anesthetized using 0.24 mg/ml of tricaine (Syndel Laboratories) and once deep anesthesia was confirmed, each fish was lightly dried, weighed using an analytical scale to determine body mass, and imaged using a light microscope (Olympus CX41, Olympus, Richmond Hill, ON, Canada) with camera system (Infinity II, Teledyne Lumenera, Ottawa, ON, Canada) to determine body length. Following recovery from anesthesia (~10 min), each fish was placed back in the housing tank. Images were scaled and measured by tracing the notochord on laterally positioned individuals on acquired images using ImageJ (www.https://imagej.nih.gov/ij/).

#### 2.3.2 Quantification of basal cortisol concentrations in larvae and adults

Pooled Larvae (n=25 per pool) from WT (n=3 replicates) and *avp ^-/-^* (n=3 replicates) were carefully collected in the morning within an hour and placed in 1.5 ml microcentrifuge tubes and stored at -80°C until processing. For adults randomly taken from mixed sex tanks, both WT (n=10) and *avp ^-/-^* (n=10) were terminally anesthetized as previously described and weighed. Blood was extracted by cutting of the dorsal fin and placed in 1.5ml microcentrifuge tubes stored at -80°C until processing. To extract cortisol from pooled larvae and adult blood samples, samples were thawed on ice and 200 μl extraction buffer was added to each tube. The samples were then sonicated, ensuring the probe was cleaned with 75% EtOH and RNAse free water in between each sample. Following homogenization, 1 ml of diethyl ether was placed into each sample, vortexed and allowed to sit for 30 min. The samples were then centrifuged at 3000 g for five min and flash frozen at -80°C for 30 min. The liquid phase was removed and placed into new microcentrifuge tubes. The ether was evaporated under a gentle stream of air under the fume hood and the extraction steps were then repeated two more times. After the final extraction step, 250 μl extraction buffer was added to the tubes, vortexed and placed in a heat block for five minutes at 65°C. The samples were vortexed, placed on the heat block for additional 5 min and vortexed for a final time. The extracts were then placed at -80°C until use. Extracts were used for the quantification of cortisol using ELISA kit #402710 (Neogen Diagnostics, Edmonton, AB, Canada) according to the manufacturer’s instructions. In all cases, samples were run in duplicates, on a single plate, and a cut-off of <20% was applied for replicates. All samples were normalized by number of larvae in each microcentrifuge tube prior to analysis.

### 2.4 Statistical analysis

For each experiment, normal distribution of data was assessed using the Shapiro-Wilk test and homoscedasticity using Levene’s test, respectively. In cases where the raw data were not parametric, standard transformations (log, sqrt, inversion, arcsin) were used to improve normality and/or homoscedasticity. Single outliers were identified in normally distributed data using Grubb’s test. For parametric and homoscedastic data, a t-test was used in case of two comparison groups and univariate ANOVAs (one-way or two-way, as appropriate) followed by Tukey’s *post-hoc* tests in cases of multiple comparison groups. In cases where data not normally distributed, Mann-Whitney U tests were used for comparisons of two groups, and Kruskal-Wallis tests for multiple groups followed by Dunn’s *post-hoc* test. In cases were the investigation of interaction of independent variables was warranted for non-parametric datasets, an ANOVA based on ranks followed by the Scheirer-Ray-Hare extension (49) was used. In all cases, a *P*-value <0.05 was considered as cut-off for significance. All analyses were conducted using SPSS Version 28 and graphs plotted using Graphpad Prism Version 9 (Graphpad Software, LaJolla, CA, USA).

## 3 Results

### 3.1 The reduced reproductive success in avp ^-/-^ fish is dependent on the female genotype

**Figure 3:**
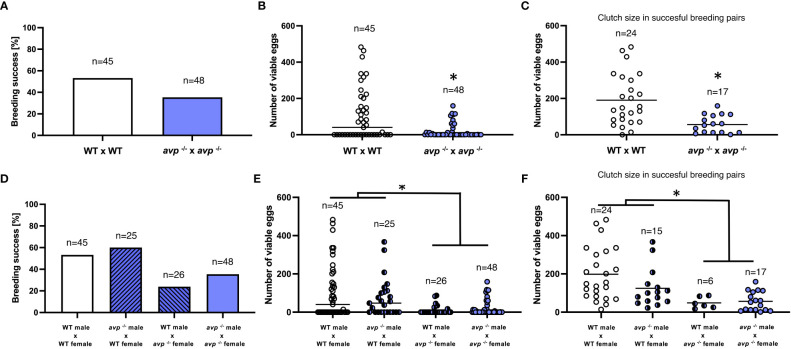
Assessment of reproductive success in WT (white bars and data points) and *avp^-/-^* (purple bars and data points) zebrafish. **(A)** Percent successful breeding trials in WT and *avp* ^-/-^ breeding pairs analyzed by Fisher’s exact test. **(B)** (Median) number of fertilized eggs in WT and *avp* ^-/-^ in all breeding trials analyzed by Mann-Whitney U test. Asterisk indicates a significant difference at *P*<0.01. **(C)** (Mean) clutch size expressed as the number of fertilized eggs in successful breeding trials analyzed by t-test. Asterisk indicates a significant difference at *P*<0.001. **(D)** Full-factorial crossbreeding experiments to delineate sex-specific contributions of the *avp* genoptype to reproductive success. Four crosses were bred: WT females x WT males; WT females x *avp ^-/-^* males; WT males x *avp ^-/-^* females; *avp* ^-/-^ males x *avp* ^-/-^ females. **(D)** (Median) number of fertilized eggs of each breeding trial across breeding groups analyzed by two-way ANOVA on ranks followed by Scheirer-Ray-Hare extension. **(E)** (Mean) Clutch size expressed as the number of fertilized eggs in successful breeding trials. Data were analyzed by two-way ANOVA **(F)**.

When comparing WT and *avp* ^-/-^ breeding pairs, the number of successful breeding was reduced from 53% in WT to 35% in *avp* ^-/-^ (**Figure 3A**). However, this reduction was not significantly different when assessed by Fisher’s exact test (*P*=0.097). Conversely, the median number of viable eggs produced was significantly reduced (Mann-Whitney U= 741.5, *P*=0.004), reaching a median of 40 in WT and 0 in *avp* ^-/-^ pairs (**Figure 3B**). When comparing average clutch size of successful breeding pairs, a significant (df=39, t=3.559, *P*=0.001), a more than 3-fold reduction was found in *avp -/-* breeding pairs compared to WT breeding pairs (**Figure 3C**). Mean clutch sizes were 190 eggs in WT and 56 in *avp ^-/-^* mutants. To delineate potential sex-specific genotype contributions, additional backcrosses between male WT and female *avp* ^-/-^ as well as female WT and male *avp* ^-/-^ were generated and the percentage of successful breeding events (**Figure 3D**), the number of viable eggs produced (**Figure 3E**) and clutch sizes (**Figure 3F**) analyzed in conjunction with data obtained from homozygous breeding pairs presented above. The median number of viable eggs produced (**Figure 3E**) depended significantly on the female (df=1 H=16.5551 *P*<0.001), but not male (df=1 H=0.320 *P*=0.572), genotype and was not dependent on the interaction of both genotypes (df=1 H=0.364 P=0.55). Regarding clutch size, a significant effect of female genotype (df=1 F=17.062; *P*<0.01), but not male genotype (df=1 F=1.550; *P*=0.218) or their interaction (df=1; F=0.99; *P*=0.754) was observed (**Figure 3F**). For the female genotypes, median number of eggs produced, and mean clutch size was significantly lower for *avp* ^-/-^ compared to WT (*P*<0.01).

### 3.2 Quivering, a courtship behavior linked to egg release is reduced in avp ^-/-^ females

**Figure 4:**
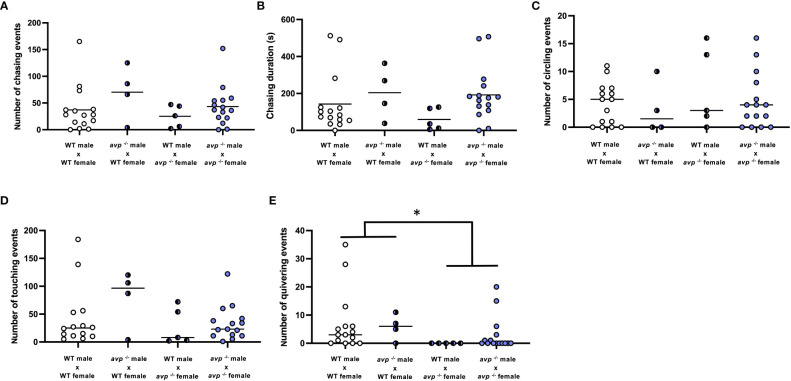
Quantification of courtship behaviors in WT and *avp ^-/-^* fish breeding pairs. The first 10 min of interaction were videotaped and analyzed by a researcher blind to the genotype of the breeding pairs. **(A)** (Median) number of nudges. **(B)** (Mean) number of chasing events. **(C)** (Mean) duration of chasing events, **(D)** (Median) number of circling events. **(E)** (Median) number of quivering events are shown. Data were analyzed by 2-way ANOVA where normally distributed or by two way-ANOVA on ranks followed by Scheirer-Ray-Hare extension for non-normally distributed data. The asterisk represents a significant effect of the maternal genotype, in which breeding pairs with *avp ^-/-^* females exhibit significantly less quivering behaviour compared to pairings with female WT.

Analysis of several aspects of courtship behaviors in breeding pairs revealed no or only marginally significant effects of male or female genotypes or their interaction. Specifically, within the first ten minutes of interaction of males and females during breeding assays, the number of male chasing events (**Figure 4A**) did not significantly depend on male genotype (df=1 F=2.571; *P*=0.118), female genotype (df=1 F=1.017 *P*=0.320), or their interaction (df=1 F=0.180 *P*=0.647). Similarly, the duration of male chasing events (**Figure 4B**) was not significantly affected by male genotype (df=1 F=4.096 *P*=0.051), female genotypes (df=1 F=1.032 *P*=0.317), or their interaction (df=1 F=0.438 *P*=0.513). The number of times males circled the females in breeding pairs (**Figure 4C**) was neither dependent on male (df=1 H=0.676 P=0.411) or female (df=1 H=0.689 *P*=0.407) genotype, nor their interaction (df=1 H=0.012 *P*=0.914). The number of times the male nudged the female’s flank (**Figure 4D**) was neither affected by male (df=1 H=1.915 *P*=0.166) nor female genotype (df=1 H=1.788 *P*=0.118) or their interaction (df=1 H=0.016 *P*=0.898). Conversely, quivering behavior was significantly affected by female (df=1 H=8.042 P<0.01), but not male (df=1 H=1.35 *P*=0.244) genotype and was significantly reduced in mating pairs with *avp* ^-/-^ females compared to WT females (**Figure 4E**). Quivering was not dependent on interaction of male and female genotypes (df=1 H=0.413 P=0.520).

### 3.3 Reduction in female reproductive success is linked to retention of mature oocytes and decreased recruitment of stage I oocytes

**Figure 5:**
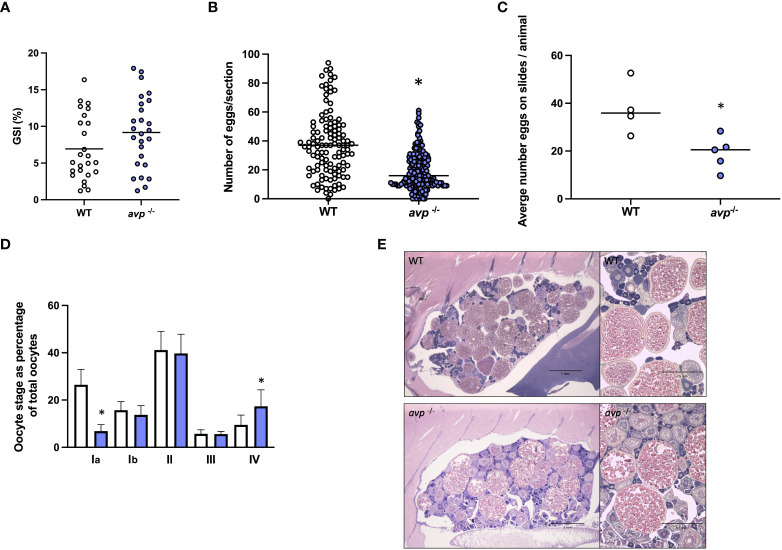
**(A)** (Mean) gonadosomatic index of WT (n=25) and *avp ^-/-^* (n=26) females. **(B, C)** (Median) total oocyte number, and oocytes staged from ovarian histology sections of WT (n=4, 120 total sections) and *avp* ^-/-^ (n=5, 150 total sections). **(D)** Representative ovarian histology sections form WT and *avp* ^-/-^ females maximum diameter measurements to determine oocyte stage distribution as (mean) percentage (± S.E.M.) of overall oocytes according to the classification system described by Li and Ge (2020). Data were analyzed by t-test in case of normal distribution and Mann-Whitney U test in case of non-normal distribution. Asterisks indicate significant differences (*P*<0.001 in **(B)**; P<0.05 in **(C, D)**. **(E)** Ovarian histology sections from gravid WT (top panel) and *avp* ^-/-^ fish (bottom panel) imaged using a stereomicroscope (left images) and light microscope respectively (right images).

The GSI was not significantly different between WT and *avp* ^-/-^ females (df=49 t=1.650 *P*=0.105; **Figure 5A**). The median number of oocytes in ovarian sections were reduced ~2-fold in *avp* ^-/-^ compared to WT sections (Mann-Whitney U=4959 *P*<0.001; **Figure 5B**). This reduction is also significant when averaging all section counts for each animal to compare WT and *avp* ^-/-^ individual animal ovaries as biological replicates (Mann-Whitney U=1 *P*=0.0317; **Figure 5C**). When comparing the distribution of oocyte stages as percentage of total oocytes within each stage (**Figure 5D**), comparisons revealed a significantly reduced percentage of stage I oocytes in *avp* ^-/-^ ovarian sections compared to WT ovarian sections (df=7 t=2.675 *P*=0.0368) and a significant increase in stage V oocytes (df=7 t=2.374 *P*=0.0493). No significant differences in the percentage of stage II (Mann Whitney U=4, *P*=0.343), III (df=3 t=1.008 *P*= 0.383) and IV oocytes (df=5.345 t=1.299 *P*=0.964) were found. Examples of stained ovary sections used for quantification and diameter measurements of oocytes in WT and *avp* ^-/-^ ovaries are shown in **Figure 5E**.

### 3.4 Ovarian PGF_2α_ is significantly reduced in avp ^-/-^ mutants

**Figure 6:**
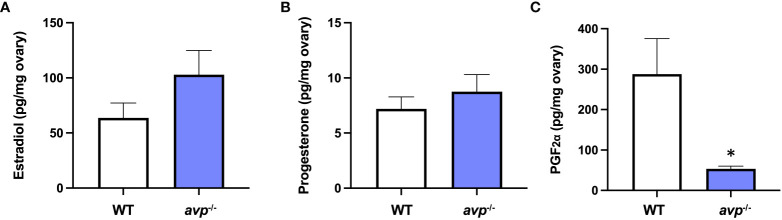
Mean ovarian sex steroid concentrations (± S.E.M.) of **(A)** E_2_, **(B)** P_4_, and **(C)** PGF_2α_ in WT (n=18) and *avp* ^-/-^ (n=18) fish. Data were analyzed by t-test and significant differences (*P*<0.05) are indicated by asterisk.

Ovarian concentrations of E_2_ (**Figure 6A**) were not significantly different between WT and *avp* ^-/-^ mutants (df=29 t=0.874 *P*=0.389). Similarly, ovarian tissue concentrations of P_4_ (**Figure 6B**) did not differ significantly between WT and *avp* ^-/-^ (df=32 t=0.6461 *P*=0.523). Conversely, PGF_2α_ (**Figure 6C**) was significantly reduced in *avp^-/-^* ovaries compared to WT ovaries (df=26 t=2.518 *P*=0.018).

### 3.5 Ovarian transcripts coding for proteins involved in PGF_2α_ synthesis are reduced in avp ^-/-^ mutants

**Figure 7:**
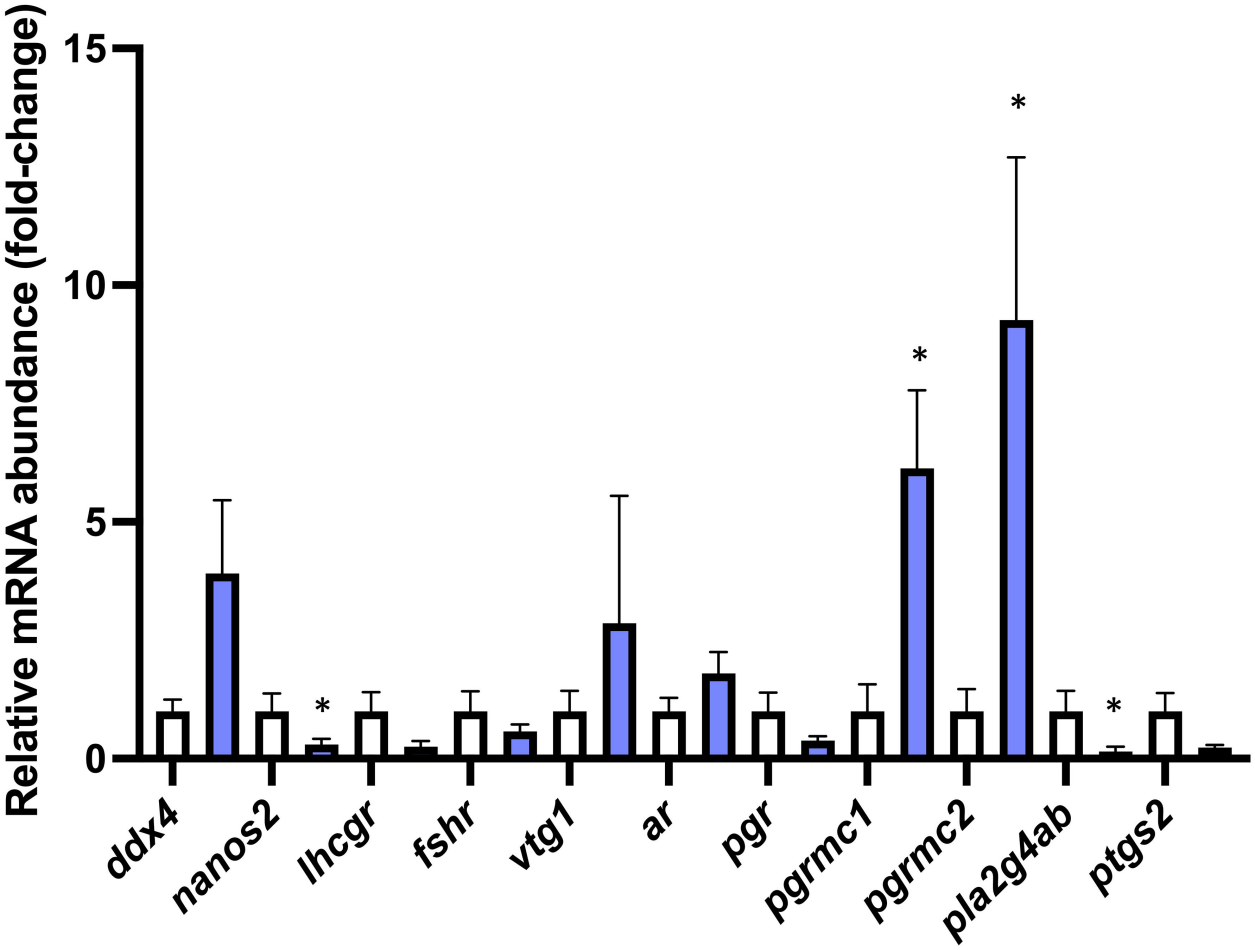
Targeted gene expression analysis of selected transcripts with characterized roles in different stages of oocyte development in WT (n=7) and *avp ^-/-^* (n=7) ovaries. Data are presented as mean values (± S.E.M.) normalized relative to WT fish transcript abundance to visualize fold-change Data were analyzed by t-test and significant differences (*P*<0.05) are indicated by asterisk.

The ovarian transcript abundances of *nanos2* (df=11 t=2.598 *P*=0.025), and *pla2g4ab* (df=12 t=3.087 *P*=0.009) were significantly reduced, while the ovarian transcript abundances of *pgrmc1* (df=7.511 t=2.809 P=0.0244) and *pgrmc2* (df=12 t=2.276 P=0.042) were significantly increased in *avp ^-/-^* mutants compared to WT (**Figure 7**). The ovarian transcript abundance of *ddx4* (df=7.403 t=0.8331 P=0.43) *lhcgr* (df=12 t=1.770 P=0.102), *fshr* (df=12 t=0.9722 *P*=0.350) *vtg1* (df=12 t=1.189 *P*=0.257), *ar* (df=12 t=1.306 *P*=0.216), *pgr* (df12 t=1.452 *P*=0.172) and *ptgs2* (df=12 t=2.027 *P*=0.066) was not significantly different between *avp* ^-/-^ mutants and WT.

### 3.6 Acute or repeated Avp injection does not attenuate the reduced female reproductive success in avp ^-/-^ mutants

**Figure 8:**
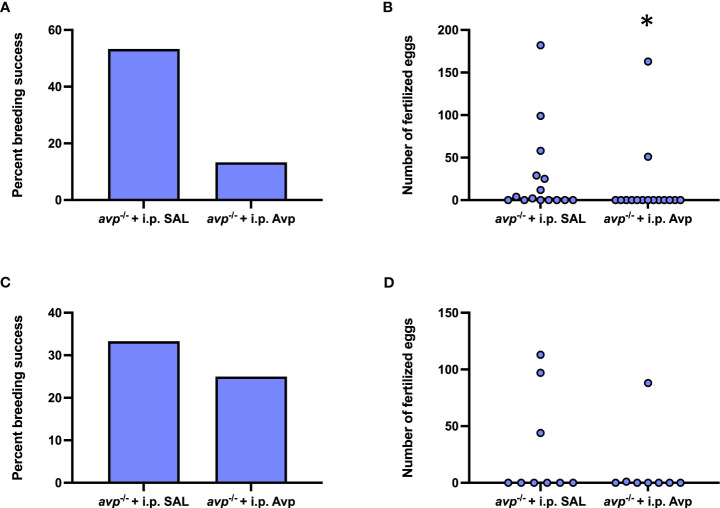
Rescue experiments assessing reproductive success in female *avp* ^-/-^ fish following single 1x acute **(A, B)** or 3x repeated i.p. injections (every 3 d) **(C, D)** of physiological saline or 1 μg/g vasotocin prior to breeding assays with WT males. For the 1x acute injection experiment, n=15 *avp* ^-/-^ females were injected with physiological saline, and n=15 with 1 μg/g vasotocin prior to breeding assays with WT males. For the 3x repeated injection experiment, n=9 *avp ^-/-^* females were injected with physiological saline, and n=8 *avp ^-/-^* females were injected with 1 μg/g vasotocin prior to breeding assays with WT males. **(A, C)** Percentage of successful breeding pairs. **(B, D)** Median number of fertilized eggs of *avp* ^-/-^ fish used in breeding assays following i.p. injection of physiological saline or 1 μg/g 1 μg/g vasotocin. Data were analyzed by Fisher’s exact test and Mann-Whitney U test, respectively, and significant differences at *P*<0.05 are indicated by asterisk.

After acute i.p. injection of *avp* -/- female prior to breeding assays, breeding success was marginally lower in Avp injected females compared saline injected females (Fisher’s exact test *P*=0.0546; **Figure 8A**) and resulted in a lower number of fertilized eggs (Mann-Whitney U=71.50 *P*=0.0442; **Figure 8B**). Repeated Avp injection in *avp* ^-/-^ compared to repeated saline-injections did not affect reproductive success (Fisher’s exact test, *P*=0.6285; **Figure 8C**) or fertilized egg number (Mann Whitney U=31 *P*=0.566; **Figure 8D**).

### 3.7 Mutant avp ^-/-^ zebrafish exhibit normal somatic growth but elevated baseline cortisol concentration

Neither body mass (df=1, F=0.27, P=0.607; **Supplemental File 1A**) nor body length (df=2, F=0.011, P=0.916; **Supplemental File 1B**) were significantly different based on genotype. Expectedly, body mass (df=1.704, F=432.164, P<0.01; **Supplemental File 1A**) and body length (df=1.658 F=310.825 P<0.01; **Supplemental File 1B**) were dependent on time, with significant increases each month up to 3 mpf (*P*>0.05). No interaction effect between genotype and time was observed for body mass (df=1.549 F=0.014 P=0.968; **Supplemental File 1A**) or body length (df=1.511 F= 0.631 P=0.508; **Supplemental File 1B**).

**Figure 9:**
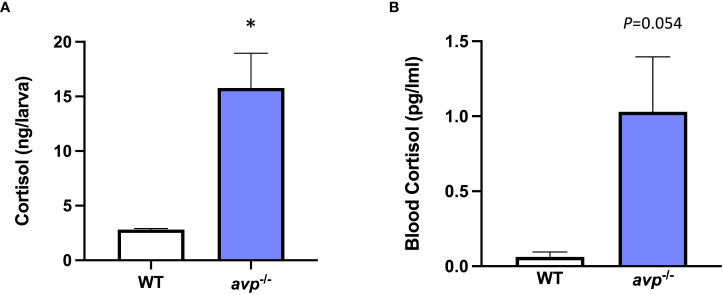
**(A)** Larval cortisol concentration at 5 dpf and **(B)** baseline cortisol concentration in sexually mature adult blood samples. Larval cortisol was determined in WT and *avp* ^-/-^ 5 dpf larvae pools (n=4 per genotype, 20 larvae per pool) according to published protocols (50). Adult whole blood cortisol was analyzed in adults of mixed sex and analyzed under baseline conditions for both WT (n=10) and *avp* ^-/-^ mutants (n=10) according to published protocols (51) with the modification that cortisol was extracted from whole blood. Data are presented as means ± S.E.M. and were analyzed using a t-test. A threshold of *P*<0.05 was used to discern a significant difference, which is indicated by asterisk.

Cortisol concentration in *avp ^-/-^* larvae was significantly higher than that of WT of controls (df=4, t=4.051, p=0.0155, **Figure 9A**) with an intra-assay coefficient of variability (CV) of 4.68%. In adults (**Figure 9B**). While cortisol concentrations in adult fish blood were elevated in *avp ^-/-^* mutant fish compared to WT, this increase was only marginally significant (*P*=0.054). An intra-assay CV of 6.68% and an inter-assay CV of 7.66% were determined for adult fish blood cortisol assays.

## 4 Discussion

### 4.1 avp ^-/-^ mutants exhibit a female-specific reduction in reproductive success

We identified a significant reduction in reproductive success in *avp* ^-/-^ breeding pairs which manifested itself by reduced egg numbers and clutch sizes, but not a significant reduction in spawning success. Backcrosses of *avp* ^-/-^ males and females with WT females and males, respectively, revealed that the observed reduction in these parameters was dependent on the female, but not male *avp* ^-/-^ genotype, as the latter reproduced similarly to WT pairs when crossed with WT females. The identification of reduced reproductive success in *avp* ^-/-^ breeding pairs compared to WT breeding pairs is in line with circumstantial evidence and mechanistic studies conducted in several teleost fish species reviewed in the introduction of this study, although these studies report effects on both female and male reproductive function, especially regarding courtship behavior (26, 27). A recent study in male zebrafish revealed that acute pharmacological inhibition of AvpR1 signaling through the i.p. injection of Manning’s compound significantly reduced reproductive success compared to saline injected males when crossed with untreated females, an effect linked to inhibition of courtship behavior (26). Similarly, the recently generated and only teleost *avp* ^-/-^ knock-out model to date, *avp* ^-/-^ Japanese medaka (27) exhibited a reduction in male dominance associated with mate-guarding, while female reproductive function was not addressed in this study. In a seasonally spawning cyprinid, the Asian stinging catfish, a suite of *in vitro* studies revealed a role for Avp in oocyte maturation by promoting germinal vesicle breakdown (GVBD), modulating steroidogenesis, and promoting ovulation (38). Early studies in natural Avp mutants in mammals (26), and recent studies of Ciona vasopressin (CiVP) in the vase tunicate, *Ciona intestinalis*, the closest existent vertebrate sister group (52, 53) suggest that a role for vasopressin on female reproductive physiology, and ovarian function in particular, may be evolutionarily deeply conserved in chordates. In the Brattleboro Long-Evans rats, an early natural mutant model characterized by the inability to produce central, but not peripheral, Avp, aberrant estrous cycle length and smaller litter size have been reported (54). In the vase tunicate, Avp stimulated oocyte maturation via promotion of germinal vesicle breakdown and ovulation via induction of metalloproteinases (52, 53).

### 4.2 Reduced female reproductive success in avp ^-/-^ mutants is linked to an inhibition of egg release

To address the underlying mechanistic basis of decreased egg release and clutch size in female *avp* ^-/-^ mutants, we investigated courtship behavior and components of the HPG axis, specifically at the level of the ovary. In contrast to previous studies demonstrating an acute role for Avp in the modulation of male courtship behaviors following pharmacological inhibition of Avp signaling via Manning’s compound, a V1aR antagonist (26), male courtship behaviors such as chasing, circling and nudging remained unaffected in any breeding combination of male or female *avp* ^-/-^ mutants and male or female WT. Such differences may be linked to the different temporal timescales in genetic and pharmacological disruption of Avp signaling, as *avp* ^-/-^ mutants may experience an alteration in organizational effects of Avp and/or compensatory effects, while acute pharmacological manipulation of receptor specific Avp signaling targets activational effects of Avp on a naturally matured Avp system. Potential organizational effects of Avp systems are only beginning to be investigated in different vertebrate species including birds (55) and mammals (56), but remain completely uncharacterized in fish. Thus, future studies are warranted to discern potential organizational an activational roles of Avp in fishes. Interestingly, quivering, a zebrafish courtship behavior linked to acute female egg release and simultaneous male milt release to maximize fertilization (57), was significantly reduced based on female, but not male *avp* ^-/-^ genotype. While not the focus of the current study, the courtship behavior also revealed a marginally significant elevation in courtship behaviors linked to the male genotype, such as for example, a tendency for increased chasing events and time, as well as touching. Future studies with increased sample size are warranted to substantiate the behavioral results and may be particularly useful to increase statistical power to fully test the possibility of behavioral consequences in *avp* ^-/-^ males compared to WT males. Interestingly, acute administration of a AvpR1 antagonist reduced these indices in a non-linear dose-dependent fashion (26), reveling that developmental knock-out of Avp produces opposite effects on indices of male courtship behavior compared to acute pharmacological modulation of the naturally matured Avp system.

In line with this female reproductive phenotype, histological analysis of *avp* ^-/-^ ovaries revealed a significantly lower quantity of oocytes in ovaries of *avp* ^-/-^ fish. Furthermore, a higher percentage of stage V oocytes was identified when quantifying oocyte stage distribution (stages I-V) as percentage of total oocytes. This finding suggests that retention of mature oocytes contributed, at least in part, to the female reproductive phenotype in *avp* ^-/-^ mutants. Because the endocrine control of the ovulation process is well known to include PGF_2α_ in zebrafish (58, 59), other cyprinids such as goldfish (60), and several other teleost fishes (61), we quantified ovarian PGF_2α_ concentrations and ovarian gene targets coding for enzymes involved in its synthesis. Indeed, ovarian concentrations of PGF_2α_ were significantly reduced by ~80% in *avp* ^-/-^ compared to WT ovaries, suggesting that Avp is a physiological regulator of mature egg release through the stimulation of PGF_2α_ in zebrafish. This is in line with detailed *in vitro* studies in another, albeit seasonally breeding fish, the Asian stinging catfish, where a suite of studies demonstrated that Avp acutely and potently stimulates PGF_2α_ synthesis in post-vitellogenic follicles (33, 38). This effect was biphasic eliciting a maximal stimulation at a concentration of 100 nM Avp which is stronger than the increase in PGF_2α_ synthesis elicited by incubation with 20 IU/ml hCG (33, 38). Concurrent incubation with Avp and hCG at aforementioned concentrations further increased ovarian PGF_2α_ synthesis, suggesting additive effects (33, 38). Finally, Avp stimulation of PGF_2α_ induced final oocyte maturation and ovulation *in vitro*, an effect that was reduced by administration of a V1aR antagonist and indomethacin, a non-selective cyclooxygenase inhibitor. In light of these studies, our findings suggest that the observed reproductive phenotype in female *avp* ^-/-^ mutants are, at least in part, mediated at the level of the ovary and that the observed roles of Avp in oocyte maturation and ovulation *in vitro* in a seasonal spawning fish extend to species with asynchronously developing oocytes such as zebrafish. It remains, however, unclear to which extent central and/or peripheral ovarian Avp systems are involved in this system and future studies using *in vitro* incubations of *avp* ^-/-^ ovarian tissue are warranted to dissect the role of the peripheral ovarian Avp system on PGF_2α_ oocyte maturation and ovulation in detail.

At the molecular level, the reduced ovarian concentration of PGF_2α_ is linked to a significant or marginally significant reduction in two key transcripts coding for enzymatic components of the PGF_2α_ biosynthesis pathway. Specifically, the transcript abundance of *pla2g4ab*, a paralogue of phospholipase A_2_, which cleaves the prostaglandin precursor arachidonic acid (AA) from membrane phospholipids (61) is significantly reduced by ~80% in *avp* ^-/-^ ovaries compared to WT ovaries. A similarly strong, albeit marginally significant, ~ 80% reduction in the transcript abundance of *ptgs2*, a paralogue coding for prostaglandin-endoperoxide synthase also known as cyclooxygenase (61). These findings suggest that ovarian Avp signaling acts to stimulate PGF_2α_ biosynthesis by inducing transcripts involved in the two regulatory steps of the PGF_2α_ biosynthesis, AA precursor mobilization and its oxidation to prostaglandin H_2_ (PGH_2_), which is further converted to mature prostaglandins including PGF_2α._ The physiological importance of both steps in ovarian zebrafish PGF_2α_ synthesis has been demonstrated in detail (58): Firstly, the importance of AA mobilization in ovarian PGF_2α_ synthesis was demonstrated by the finding that increasing concentrations of AA dose-dependently stimulate PGF_2α_ irrespective of ovarian follicle stage (58). Secondly, the importance of ovarian cyclooxygenase in ovarian PGF_2α_ in zebrafish ovarian follicles *in vitro* was demonstrated by the fact that indomethacin, a non-selective cyclooxygenase 2 inhibitor significantly reduced AA stimulated PGF_2α_ synthesis in vitellogenic follicles *in vitro*. Conversely, no changes were observed in ovarian E_2_ and P_4_, which is in contrast of reported regulation of both steroids by Avp in oocytes of a seasonal breeder the Asian stinging catfish (38). However, because season-dependent stimulatory and inhibitory effects of Avp on these sex steroids were observed in catfish oocytes, it is possible that ovarian cell specific effects were masked in asynchronous ovaries of zebrafish, which contain multiple oocyte maturation stages at a given point in time.

Because ovarian PGF_2α_ released during ovulation into female circulation is a potent regulator of female reproductive behavior in several (cyprinid) fishes (60, 62, 63) which through secretion via urine and action as a pheromone furthermore elicits male courtship to synchronize mature gamete release (47, 64, 65), it is feasible that the described reduction in quivering behavior in female *avp* ^-/-^ mutants is a consequence of reduced Avp-dependent PGF_2α_ release. Future i.p. PGF_2α_ rescue studies in *avp* ^-/-^ mutant females are warranted to discern which aspects of the reproductive phenotype observed in female *avp* ^-/-^ are dependent on disrupted ovarian PGF_2α_ synthesis.

### 4.3 Additional aspects of the female reproductive phenotype in avp ^-/-^ mutants

In addition to the described integrative evidence suggesting that female reproductive consequences of *avp* ^-/-^ knockouts are, at least in part, dependent on Avp-dependent stimulation of ovarian PGF_2α_, our mechanistic studies point to additional, previously unidentified mechanisms. When considering the distribution of oocyte stages as percentage of total oocytes in *avt* ^-/-^ compared to WT, a significantly lower percentage of stage I oocytes are observed in *avp* ^-/-^ compared to WT ovarian sections. This suggests that Avp may, in addition to previously discussed roles in oocyte maturation and ovulation, play a role in germ line stem cell maintenance, proliferation and/or differentiation into stage I follicles. In zebrafish, and in contrast to mammals which possess a finite number of oocytes following embryogenesis, zebrafish can produce new oocytes throughout their lifetime due to the presence of self-renewing germline stem cells (GSCs) (66). Indeed, the possibility of Avp effects on this system is supported by the finding that transcripts of *nanos2* are significantly reduced in *avp* ^-/-^ ovaries compared to WT ovaries. The *nanos2* transcript encodes an RNA-binding protein that is specifically expressed in zebrafish GSCs (67). In a recent detailed single-cell RNA-seq analysis of ovarian cells in zebrafish, it has specifically been shown to be present in a single, temporally early subcluster of GSC, which is in contrast to another GSC marker, *ddx4* (formerly *vasa*), which is present in all nine identified subclusters of GSC as well as stage 1A oocytes (66). Such detailed single cell expression analyses of ovarian cell populations in zebrafish provide an explanation for the observed discrepancy in GSC marker expression observed in *avp* ^-/-^ compared to WT ovaries, as expression of *ddx4*, an ATP-dependent RNA helicase identified in zebrafish GSCs across development (68) did not change significantly between genotypes and revealed a tendency for an increase in transcript abundance in *avp* ^-/-^ compared to WT. Together, these findings may suggest a specific reduction of early pool GSC in the absence of Avp in the zebrafish ovary. While functionally *nanos2* has been shown to maintain ovarian GSCs and to drive zebrafish ovarian regeneration following ablation (66), it is currently unknown whether *nanos2*-positive GSC are directly responsive to Avp viaexpression of receptors or whether indirect effects manifested in the *avp* ^-/-^ ovaries, such as the retention of final stage oocytes and/or a reduction in ovulation are involved in this phenotype. Interestingly, using the searchable single cell RNA-seq database published in (66), *avp*, *avpr1ab* and to. Lesser extent *avpr2ab* are while not highly abundant, mostly expressed in GSCs, and more specifically, early GSCs, to which *nanos2* expression is restricted (**Supplemental File S2**). Thus, it is possible that Avp directly regulates a specific pool of undifferentiated GSCs in zebrafish. This possibility clearly warrants further investigation, as regulation of (ovarian) GSCs by Avp have not been described in any species.

Finally, ovarian gene expression analysis revealed a significant increase in progestin receptor membrane components 1 and 2 transcripts (*pgrmc1*, *pgrmc2*), which code for single transmembrane heme-binding protein containing a cytochrome b5 (Cytb5) motif and which have been proposed to function as a progesterone binding protein in vertebrates, while also interacting with various molecules such as heme, fatty acid 2-hydroxylase, cytochrome P-450 enzymes, insulin receptor, epidermal growth factor receptor (EGFR), Erbb2, and membrane progestin receptor α (mPRα), among others. Thus, its precise physiological roles difficult to dissect (69). In female zebrafish, *pgrmc1 and pgrmc2* are most abundantly expressed in ovarian tissue, with a reported expression peak during oogenesis in stage I follicles (69, 70). Recent single cell sequencing of ovarian cells in mature females provided a higher resolution of cell-type sand stage specific expression profiles, revealing a more widespread expression in ovarian cells with the highest expression found located in stromal and thecal cells for *pgrmc1* and differentiating GSCs and follicles for *pgrmc2* (66). Irrespective of distribution, global functional roles in ovaries have been assessed for both *pgrmc1* and *pgrmc2* using knock-out approaches in zebrafish *in vivo* as well as pharmacological approaches in oocytes *in vitro* (69–71). These studies revealed, among other effects, a role for Pgrmc in the promotion of meiotic maturation in oocytes via stabilization of mPr and thus progestin sensitivity *in vivo* and *in vitro* (69). Thus, while the precise role of Pgrmc1 and Pgrmc2 is difficult to disentangle due to the widespread expression in ovarian cell types (66), it is possible that the observed increase in matured oocytes observed in *avp ^-/-^* ovaries compared to WT is partially dependent on the promotion of meiotic oocyte maturation in zebrafish.

### 4.4 Potential role of pleiotropic effects of Avp on the female reproductive phenotype in avp ^-/-^ mutants

Because our study investigated the reproductive consequences of Avp using our newly created *avp* ^-/-^ mutant *in vivo*, it is important to at least consider the possibility of indirect pleiotropic effects of Avp. In teleost fishes, several physiological roles for Avp have been described, including, but not limited to, the regulation of energy balance, the regulation of the endocrine stress axis, osmoregulation and cardiovascular effects, as well as circadian regulation (72). While it is impossible to assess all phenotypic characteristics in the *avp* ^-/-^ knock out model in the context of this study, we elucidated potential consequences on somatic growth, a more important determinant of reproductive maturity in developing female zebrafish compared to age (73). While potent effects of Avp on the regulation of feed-intake have been described in adult teleost fishes (74), no difference in body mass or body length was found between *avp* ^-/-^ and WT fishes up to sexual maturity 3 mpf, suggesting that the observed reproductive effects are not secondary effects linked to overt differences in somatic growth until achieving sexual maturity. Secondly, because Avp has been characterized to be involved in the regulation of the endocrine stress axis in teleost fishes (72, 74), we assessed larval and adult cortisol concentrations in *avp ^-/-^* mutants and WT. Both larvae and adult zebrafish exhibited an elevated concentration of cortisol, raising the possibility that hypercortisolism may contribute the described female reproductive phenotype in *avp* ^-/-^ mutants. Increased cortisol has been shown to affect zebrafish ovarian function and oogenesis *in vitro* and *in vivo* (75–77). Briefly, direct incubation of stage I and especially stage II follicles with 1 μM cortisol induced increased DNA damage as assessed by comet assay (75). In female *gr* ^-/-^ zebrafish characterized by hypercortisolism, an increase in oocyte cortisol and a reduction in ovulated eggs has been identified (76). Unfortunately, no oocyte stage distribution has been reported in this study (76). In females of another *gr* ^-/-^ knock-out model, increased fecundity was observed in early adult females, while decreased fecundity was observed in adult females, indicative of early ovarian senescence (77). In both age-groups, increased follicular atresia was observed. However, especially because a buffering capacity with regard to cortisol load that is mediated via induction of 11β-hydroxysteroid dehydrogenase type 2 (*11β-hsd2)*, a cortisol inactivating enzyme, has been described in zebrafish oocytes (78), additional studies are necessary to specifically address potential contributions of cortisol to the observed reproductive phenotype in female *avp* ^-/-^ fish.

## 5 Conclusion

In sum, our study using newly created *avp* ^-/-^ knock-out zebrafish shows that reproductive function, as quantified egg release and clutch size, is significantly reduced and that this reduction is dependent on female mutants. Integrative mechanisitic investigation of the underlying mechanisms of this phenotype strongly suggest that this phenotype is at least in part dependent on reduced ovulation mediated by a disrupted PGF_2α_ synthesis, which may also underlie the reduction in quivering behavior associated with ovulation. These findings are in line with previous findings from *in vitro* studies investigating the role of Avp on oocytes maturation and function in asynchronously spawning catfish (38), and contribute to the emerging larger picture of an evolutionary conserved role for Avp peptides in oocyte maturation and release in chordates (52). Mechanistically, our study additionally uncovers a potentially new mechanism for Avp on oocyte maturation, revealing the possibility of direct regulation of undiffertitated *nanos2+* pools of GSCs by Avp which merit further investigation. Because no rescue of the *avp* ^-/-^ female reproductive phenotypes was found following acute and repeated Avp administration, it is suggested that potential organizational effects of Avp on ovarian development should be further investigated. In the rescue experiment, saline i.p injection in WT did not result in differences in percent breeding success or median numbers of fertilized eggs produced compared to WT breeding pairs used in breeding assays, suggesting that the i.p. injection stress is negligible or does not exert an acute effect on female egg release, at least in WT fish. In *avp* ^-/-^ mutants, characterized by elevated baseline cortisol concentrations, however, it is possible that the i.p. injection may elicit a qualitatively different stress response with secondary effects on the egg release. While we did not quantify cortisol concentrations in the rescue experiments, we believe that the fact that fewer oocytes were identified in *avp ^-/-^* females compared to WT females not used in breeding assays, future studies should focus on potential organizational roles of Avp in ovarian development.

## Data availability statement

The raw data supporting the conclusions of this article will be made available by the authors, without undue reservation.

## Ethics statement

The animal study was reviewed and approved by University of Ottawa Animal Care Committee.

## Author contributions

DR, VS and JM contributed to conception and design of the study. VS and DR generated the knock-out line, DR and KS conducted experiments. DR and JM performed the statistical analysis. DR, KS, VS and JM wrote the first draft of the manuscript. NN and DCR contributed to the acquisition and analysis of data (histology), critical revisIon and of the manuscript, final approval of the submitted manuscript, agreement to be accountable to all aspects of the work. All authors contributed to manuscript revision, read, and approved the submitted version.

## Supplementary material

Supplemental File S1 Developmental trajectories of **(A)** body mass, **(B)** body length, and **(C)** calculated BMI, in WT and *avp* ^-/-^ zebrafish. Developing zebrafish WT (n=20); *avp* ^-/-^ (n=20) were weighed and their body length measured monthly until reaching sexual maturity at 3 mpf. All data are presented as mean ± S.E.M. and were analyzed using repeated measurement ANOVAs.

Supplemental File S2
*In silico* analysis of expression of *avp* and detected receptors (*avpr1ab*, *avpr2ab*) as well as *nanos2* in all ovarian cell types and differentiation stages (top row) and differentiating GSCs (bottom row) as described by single cell sequencing of 40 dpf female zebrafish ovaries (66). The database (https://singlecell.broadinstitute.org) was accessed on Sep 5^th^, 2022. The following manual annotations were added: GSC - germinal stem cells; EOO - early oogenesis; THE - thecal cells; FOL - follicles; STR - stroma cells; VAS - vasculature; IMM - immune cells; MEI - meiotic division stage.
